# Complete mitochondrial genomes of eight seahorses and pipefishes (Syngnathiformes: Syngnathidae): insight into the adaptive radiation of syngnathid fishes

**DOI:** 10.1186/s12862-019-1430-3

**Published:** 2019-06-11

**Authors:** Xin Wang, Yanhong Zhang, Huixian Zhang, Geng Qin, Qiang Lin

**Affiliations:** 1 CAS Key Laboratory of Tropical Marine Bio-Resources and Ecology, South China Sea Institute of Oceanology, Institution of South China Sea Ecology and Environmental Engineering, Chinese Academy of Sciences, Guangzhou, 510301 People’s Republic of China; 2 Laboratory for Marine Fisheries Science and Food Production Processes, Pilot National Laboratory for Marine Science and Technology (Qingdao), Qingdao, 266237 People’s Republic of China; 3 University of the Chinese Academy of Sciences, Beijing, 100049 People’s Republic of China

**Keywords:** Syngnathidae, Seahorse, Pipefish, Evolution, Purifying selection, Mitogenome

## Abstract

**Background:**

The evolution of male pregnancy is the most distinctive characteristic of syngnathids, and their specialized life history traits make syngnathid species excellent model species for many issues in biological evolution. However, the origin of syngnathids and the evolutionary divergence time of different syngnathid species remain poorly resolved. Comprehensive phylogenetic studies of the Syngnathidae will provide critical evidence to elucidate their origin, evolution, and dispersal patterns.

**Results:**

We sequenced the mitochondrial genomes of eight syngnathid species in this study, and the estimated divergence times suggested that syngnathids diverged from other teleosts approximately 48.8 Mya during the Eocene period. Selection analysis showed that many mitochondrial genes of syngnathids exhibited significantly lower *Ka/Ks* values than those of other teleosts. The two most frequently used codons in syngnathid fishes were different from those in other teleosts, and a greater proportion of the mitochondrial simple sequence repeats (SSRs) were distributed in non-coding sequences in syngnathids compared with other teleosts.

**Conclusions:**

Our study indicated that syngnathid fishes experienced an adaptive radiation process during the early explosion of species. Syngnathid mitochondrial OXPHOS genes appear to exhibit depressed *Ka/Ks* ratios compared with those of other teleosts, and this may suggest that their mitogenomes have experienced strong selective constraints to eliminate deleterious mutations.

**Electronic supplementary material:**

The online version of this article (10.1186/s12862-019-1430-3) contains supplementary material, which is available to authorized users.

## Background

Members of the teleost family Syngnathidae (seahorses, pipefishes, pipehorses, and seadragons), comprising approximately 300 species, are unique among vertebrates in that they exhibit ‘male pregnancy’, where males incubate developing embryos in a brood pouch until hatching and parturition [1–3]. The specialized brood pouch provides protection and carries out gas exchange, osmoregulation, and limited nutrient provisioning for the developing embryos [4, 5]. In addition, members of the family Syngnathidae exhibit other unique features such as an extended body covered with an armor of bony plates instead of scales, elongated snout, and fused jaws. The unique body morphology and specialized life history traits make syngnathid species excellent flagship species for many issues in marine conservation and biological evolution [4, 6].

There has been considerable research on the phylogeny, life history and biological characteristics of syngnathid fishes [4, 7–12]. However, the biological origin and divergence history of syngnathids still unclear. The family Syngnathidae is a large and diverse clade of bony fishes, and male brood-pouch morphology was a major focus of previous evolutionary research. The brooding structures vary greatly between genera, from the simplest incubating area typical of the Nerophinae to much more complex structures, such as the sealed pouch of the Hippocampinae [13]. Previous studies hypothesized that syngnathids can be divided into five major subfamilies based on brood pouch morphology, and subsequent studies divided syngnathids into two large clades based on the position of the male brood pouch [3, 12–15]. These phylogenetic divisions have been supported in molecular phylogenetic analyses based on partial mitochondrial sequences [13, 15, 16]. However, the reliability of the hypothesis has not yet been confirmed using more representative data. To date, few studies have investigated the divergence times of the different syngnathid lineages, and this information will provide critical evidence to elucidate the origin, evolution, and dispersal patterns of the family Syngnathidae.

Mitochondrial genomes have been widely used for diverse evolutionary studies of animals, including population genetics, phylogenetics, and species identification [13, 17–20]. The circular mitogenome of teleosts is structurally conserved and contains 13 protein-coding genes, 2 rRNA genes, 22 tRNA genes, and one displacement loop (D-loop) region [21, 22], that can provide a large amount of basic data for population genetics, phylogenetics, and adaptive evolution research [13, 23]. More than one thousand complete mitochondrial DNA sequences have been determined in teleostean fishes [24], but systematic research on mitogenome structure and molecular evolution characteristics in syngnathids is still scarce. Previous studies observed gene reorganization in fish mitogenomes [24–26], and recent studies have shown that the accumulation of mutations in mitogenomes is influenced by life history, effective population size, and cellular energy requirements [27–30]. Given the specialized biological characteristics and extraordinary evolutionary status of syngnathid fishes, we suspected that the structure and molecular evolution characteristics of their mitochondrial genomes may exhibit significant differences compared with those in other teleosts.

In this study, the mitogenomes of eight syngnathid species were assembled to investigate the phylogenetic relationships and divergence times of syngnathid lineages. We also obtained the mitochondrial genome sequences of a further 88 teleost species for comparison of the structure and molecular evolution characteristics between syngnathids and other teleosts.

## Results

### Mitochondrial genomes in the Syngnathidae

**Fig. 1:**
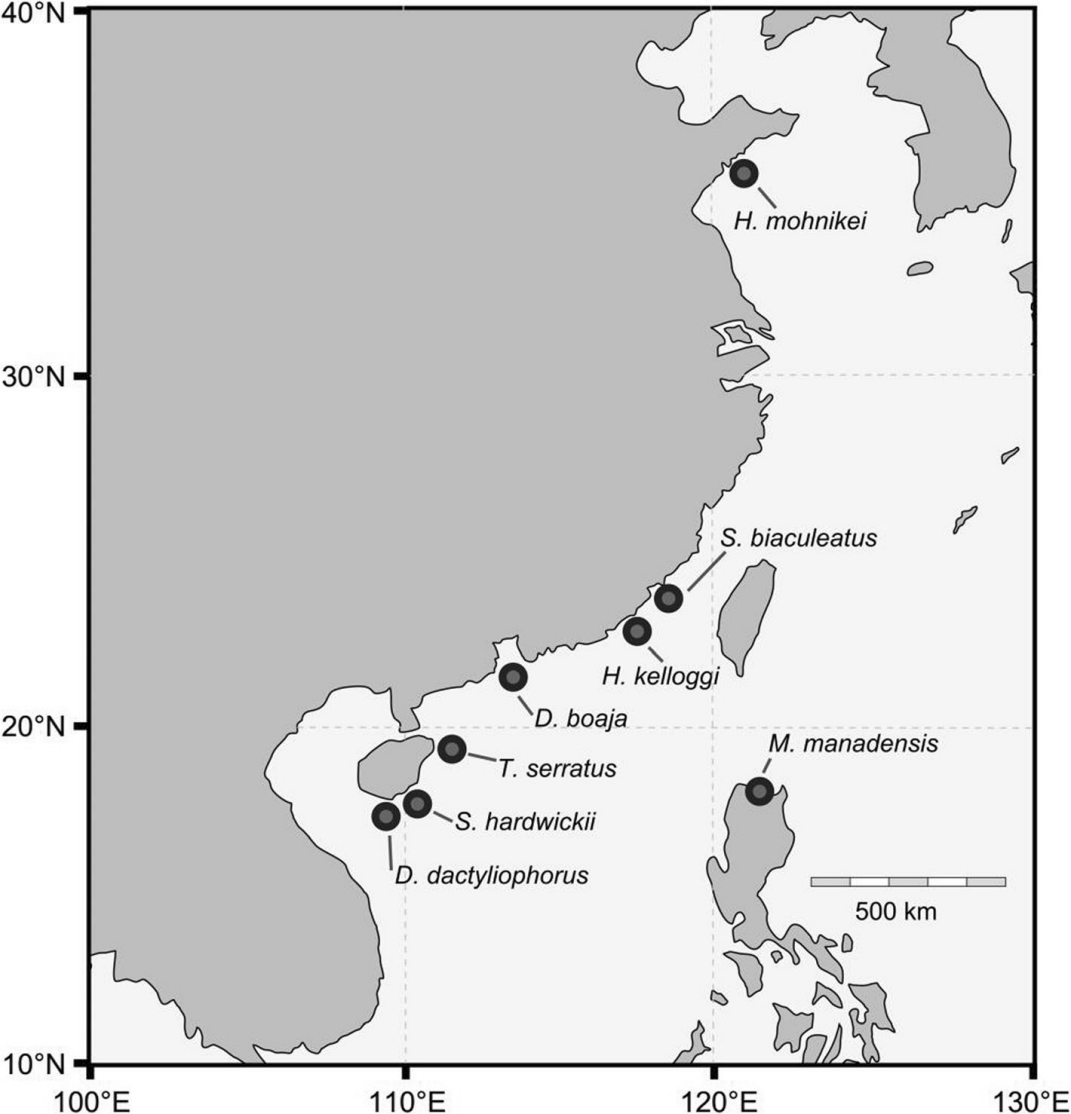
Map of sampling locations for syngnathid fishes

**Fig. 2:**
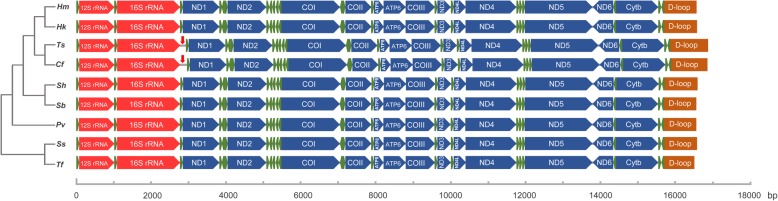
Linear comparison of mitochondrial genome organization in syngnathid fishes and other teleosts. Hm, *Hippocampus mohnikei*; Hk, *Hippocampus kelloggi*; Ts, *Trachyrhampus serratus*; Cf, *Corythoichthys flavofasciatus*; Sh, *Solegnathus hardwickii*; Sb, *Syngnathoides biaculeatus*; Pv, *Pegasus volitans*; Ss, *Salmo salar*; Tf, *Takifugu flavidus*

The complete mitochondrial genomes of the syngnathid species ranged in size from 16,462 bp to 16,961 bp, with the newly determined *T. serratus*, *S. hardwickii*, *S. biaculeatus*, *D. boaja*, *D. dactyliophorus*, *M. manadensis*, *H. kelloggi*, and *H. mohnikei* mitochondrial genomes exhibiting lengths of 16,956 bp, 16,519 bp, 16,479 bp, 16,547 bp, 16,661 bp, 16,527 bp, 16,536 bp, and 16,518 bp, respectively (Fig. 1, Table 1). The differences in genome length were largely due to variations in tandem repeats in the control regions. An approximately 200 bp non-coding insertion between 16S-rRNA and tRNA-Leu was found in *T. serratus* and *C. flavofasciatus* (Fig. 2). All the genomes shared 13 protein-coding genes, 22 tRNA genes, 2 rRNAs, and a control region, and exhibited the same gene order (Fig. 2). The AT content of the mitogenome ranged from 55.31 to 62.07% for the eight newly sequenced species, with a slight AT bias.

**Table 1:** Genome information of the newly sequenced mitochondrial genomes of 8 syngnathid fishes

Species	Accession Numbers	Size (bp)	Nucleotide composition (%)	A + T (%)	AT skewness	GC skewness
*Trachyrhamphus serratus*	KJ184528	16,956	A 29.61 C 28.35 G 16.34 T 25.70	55.31	0.071	−0.269
*Solegnathus hardwickii*	KJ194524	16,519	A 29.72 C 28.39 G 15.16 T 26.73	56.45	0.053	−0.304
*Syngnathoides biaculeatus*	KJ184525	16,479	A 29.69 C 26.56 G 15.38 T 28.37	58.06	0.023	−0.267
*Doryichthys boaja*	KJ184527	16,547	A 31.12 C 24.10 G 14.37 T 30.41	61.53	0.012	−0.253
*Dunckerocampus dactyliophorus*	KP301502	16,661	A 30.05 C 27.89 G 16.04 T 26.02	56.07	0.072	−0.270
*Microphis manadensis*	KP301501	16,527	A 30.28 C 26.15 G 15.20 T 28.37	58.65	0.033	−0.265
*Hippocampus kelloggi*	KF703755	16,536	A 32.19 C 23.69 G 14.81 T 29.31	61.50	0.047	−0.231
*Hippocampus mohnikei*	KF557651	16,518	A 32.10 C 22.92 G 15.01 T 29.97	62.07	0.034	−0.209

### In silico analysis of simple sequence repeats (SSRs) in mitochondrial genomes

**Fig. 3:**
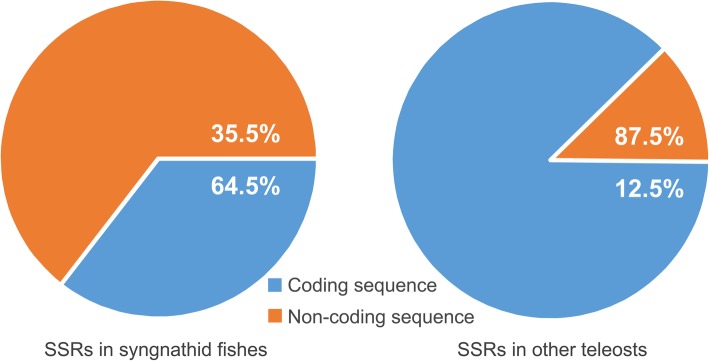
Distribution of SSRs in the mitogenome of syngnathid fishes and other teleosts

A total of 64 SSRs of different nucleotide combinations were detected in 44 species. An equal number of SSRs were detected in the syngnathids and the other teleosts: 32 SSRs were found in 18 of the 22 syngnathids, and 32 SSRs were found in 19 of the 22 other teleosts (Additional file 1). However, the distribution patterns of the SSRs were very different between the syngnathids and the other teleosts. In the syngnathids, 35.5% of the SSRs were detected in coding sequences and 64.5% in non-coding sequences. In contrast, 87.5% of the SSRs were found in coding sequences in the other teleosts, and only 12.5% of the SSRs were distributed across non-coding sequences (Fig. 3). Overall, a greater proportion of the mitochondrial SSRs were distributed in non-coding sequences in syngnathids compared with other teleosts.

### Nucleotide diversity and codon usage

**Fig. 4:**
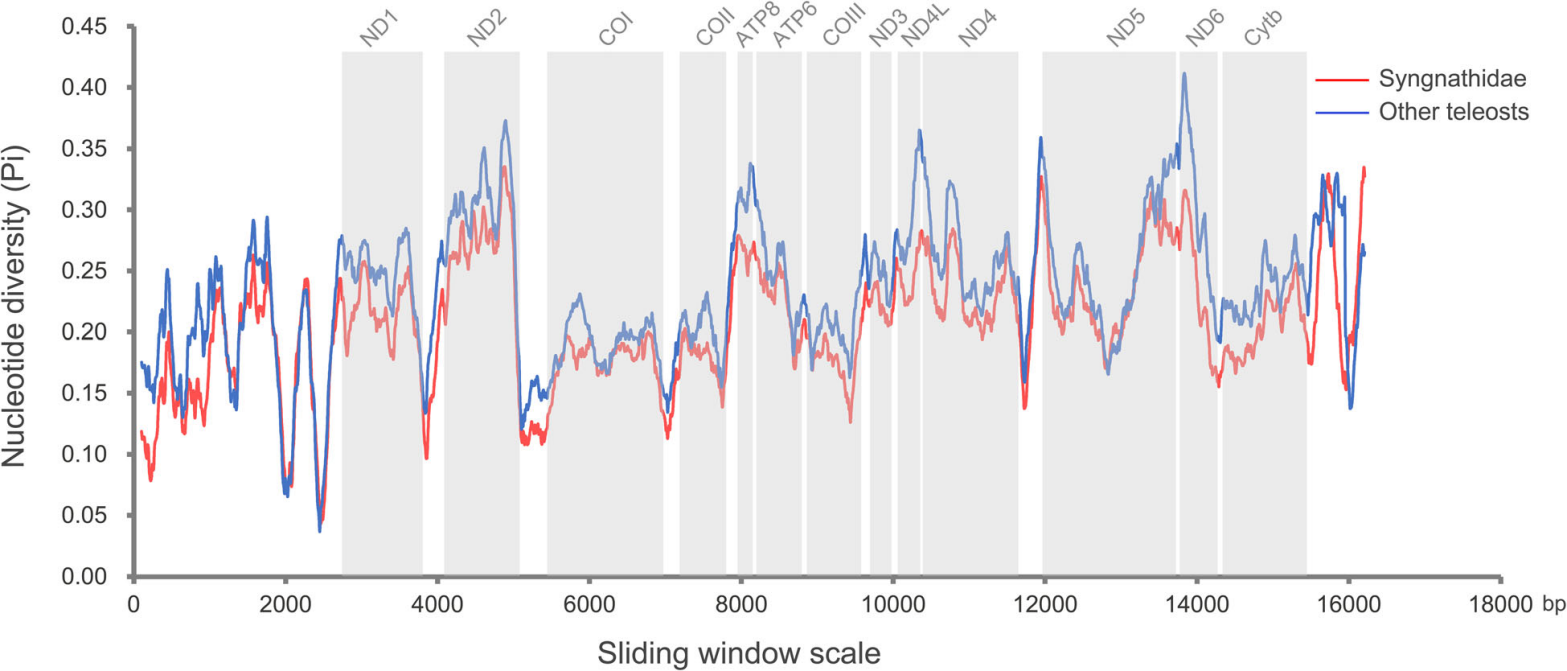
Nucleotide diversity (Pi) of the mitochondrial genome sequences of syngnathid fishes and other teleosts

Sliding window analysis of sequences from 22 syngnathids and 22 other teleosts was employed to evaluate the nucleotide diversity of the mitogenomes. The nucleotide diversity exhibited similar patterns in the two groups, with Pi values ranging from 0.043 to 0.335 in syngnathids and 0.037 to 0.412 in other teleosts (Fig. 4). The *ND2*, *ND6*, and *ATP8* regions showed relatively high sequence variability, while genes with relatively low sequence variability included *COI*, *COII*, *COIII*, and *Cyt b* (Fig. 4).

**Fig. 5:**
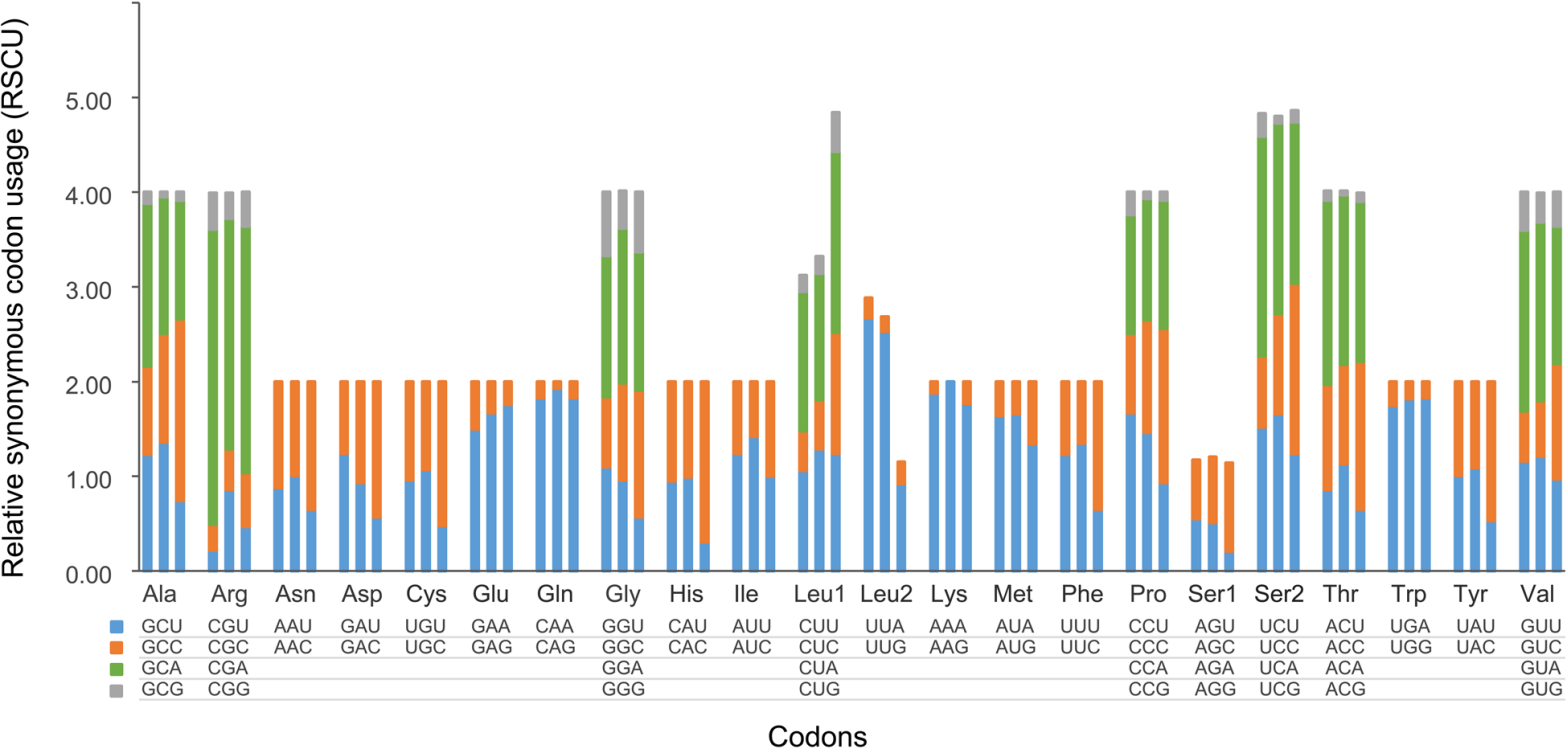
Relative synonymous codon usage (RSCU) of the mitochondrial genomes. Left, *Hippocampus mohnikei*; middle, *Doryichthys bojia*; right, *Takifugu flavidus*

A significant bias towards A/T was observed in the codon usage of the mitochondrial genomes of the syngnathids *Hippocampus mohnikei* and *Doryichthys bojia*, and the other teleost *Takifugu flavidus* (Fig. 5, Additional file 2), and G was the least common third position nucleotide in all the codon families. The most frequently used codons in *H. mohnikei* and *D. bojia* were UUA (Leu) and AUU (Ile), while the most frequently used codons in *T. flavidus* were UUC (Phe) and CUA (Leu) (Additional file 3). The most frequently used codons all consisted of A and T, and this may have contributed to the high A + T content in the mitogenome. There was an obvious difference between the two syngnathid fishes and *T. flavidus* in relative synonymous codon usage (RSCU), especially in Cys, His, Leu, and Phe. Among the 62 available codons (excluding TAA and TAG), AGA (Arg) was missing in *H. mohnikei*, *D. bojia*, and *T. flavidus*, while AGG (Arg) was missing in *H. mohnikei* and *D. bojia* (Fig. 5, Additional file 4).

### Phylogenetic reconstruction and divergence time estimation

**Fig. 6:**
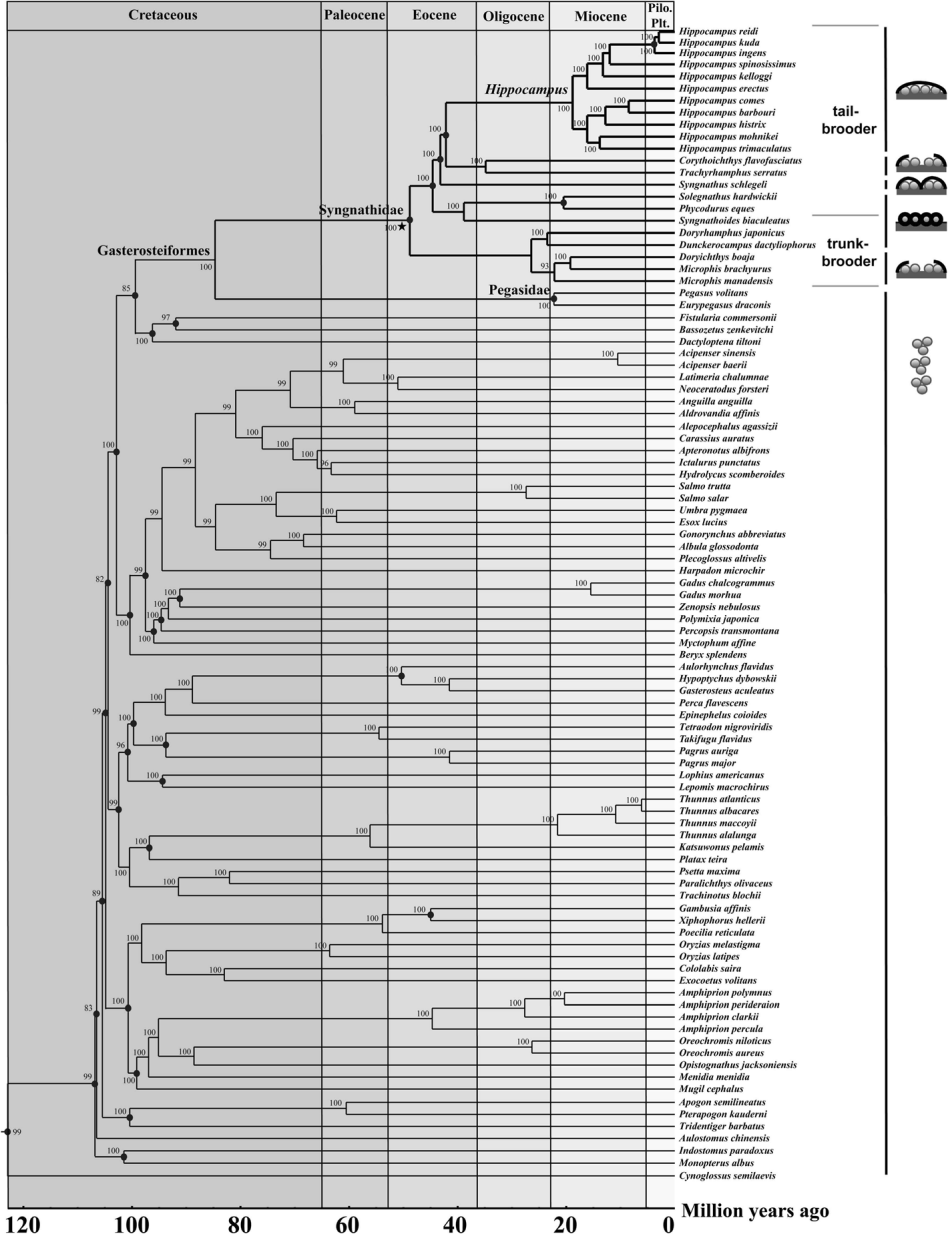
Time-calibrated phylogeny of 96 fishes based on 13 mitochondrial genes. The black dots represent calibration points, and the five-pointed star represents a seahorse fossil. Cross-sections of the brood pouch:, fully enclosed pouch;, enclosed pouch (inverted);, rudimentary pouch with plates and skin-folds;, individual egg compartments;, unprotected eggs (free spawning)

The phylogenetic relationships between syngnathids and other teleosts were determined using 13 mitochondrial gene sequences. The best substitution model for the data matrix was GTR + G + I, and maximum-likelihood (ML) analyses yielded a well-resolved phylogeny for the fishes: most of the internal branches were supported by high (> 90%) bootstrap probabilities (BPs; Fig. 6). The Syngnathidae family is a unique clade of bony fishes that exhibit special male brooding structures and our phylogenetic analysis recovered the syngnathids as a monophyletic group, with a high level of confidence (99% BP). The divergence times of syngnathids and other teleosts estimated using the MCMC tree suggest that syngnathids diverged from the other teleosts approximately 48.8 Mya (95% CI 48.4–49.0 Mya), during the Eocene period. Eleven *Hippocampus* species formed a monophyletic clade which constituted the largest genus (BP = 100%) that appeared 18.8 Mya (95% CI 13.7–24.0 Mya, Additional file 3), during the Miocene period. Phylogenetic reconstruction suggested that *Corythoichthys flavofasciatus* is the closest living relative of *Trachyrhamphus serratus* and a sister clade to *Hippocampus*. Pegasidae and Syngnathidae were the closest sister groups (BP = 100%). Indostomidae clustered with *Monopterus albus* (Synbranchiformes). However, Gasterosteoidei did not cluster with Syngnathoidei. The gasterosteoids *Aulorhynchus flavidus*, *Gasterosteus aculeatus*, and *Hypoptychus dybowskii* formed a group in the ML tree parallel to *Epinephelus coioides* (Perciformes).

**Fig. 7:**
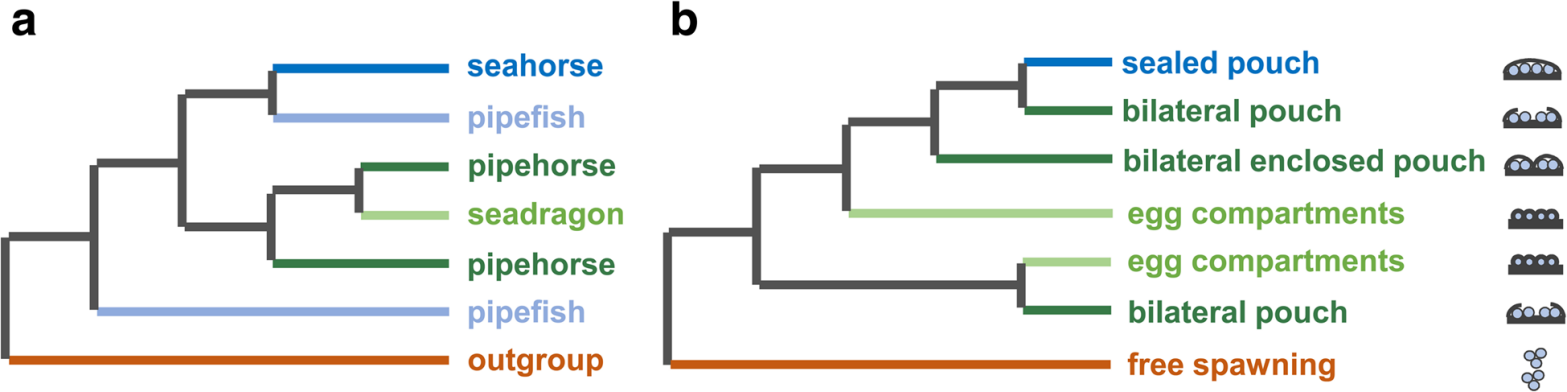
Schematic diagram of the phylogenetic relationships of syngnathid groups (**a**), and phylogenetic analysis of the syngnathids with various brooding structures (**b**). Cross-sections of the brood pouch, fully enclosed pouch;, enclosed pouch (inverted);, rudimentary pouch with plates and skin-folds;, individual egg compartments;, unprotected eggs (free spawning)

The schematic diagram of the phylogenetic relationships of syngnathid groups shows that the pipefish can be divided into two genetic lineages; one clustered with seahorses, and the other clustered independently of other syngnathids (Fig. 7a). Additionally, the phylogenetic analysis revealed that the pipehorses were paraphyletic with respect to seadragons (Fig. 7a). Phylogenetic analysis of the syngnathids with various brooding structures revealed asynchronous evolution of the brood pouch. The seahorses with a sealed pouch formed a monophyletic group, and this clustered with one lineage of bilateral-pouch syngnathids, while the other lineage of bilateral-pouch syngnathids clustered with syngnathids that have no pouch structure but egg-compartments on the abdomen (Fig. 7b).

### Male-pregnant syngnathids are under strong purifying selection

**Fig. 8:**
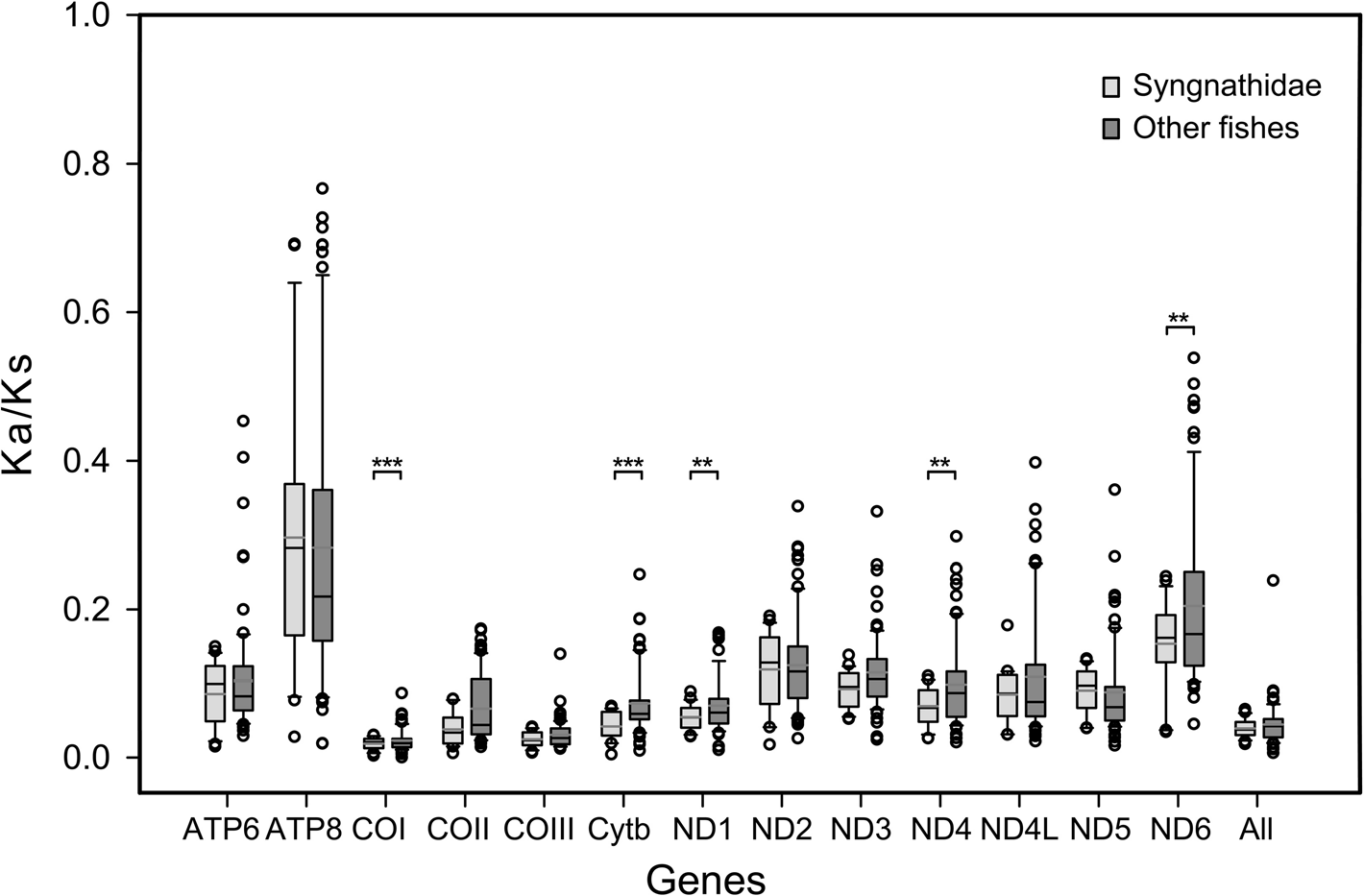
Comparison of *Ka/Ks* ratios for 13 individual genes and 13 gene sets between syngnathids and other fishes. “All” indicates the combined sequences of the 13 mitochondrial genes. The asterisks indicate the significance of the likelihood ratio test results from the free-ratio and one-ratio models (*, 0.01 < *P* < 0.05; **, 0.001 < *P* < 0.01; ***, *P* < 0.001)

Differences in the *Ka/Ks* ratios of fishes with different reproductive strategies were quantified by separate analyses of two groups: the male-pregnant syngnathid fishes and other teleostean fishes. The mean *Ka/Ks* ratio of all mitochondrial genes was slightly lower in the male-pregnant syngnathids (*Ka/Ks =* 0.0392) than in the other teleostean fishes (*Ka/Ks =* 0.0445; Fig. 8, Additional file 4). Investigation of the general pattern of *Ka/Ks* in the 13 mitochondrial protein-coding genes showed that five genes (*COI*, *Cyt b*, *ND1*, *ND4*, and *ND6*) exhibited significantly lower *Ka/Ks* values in the syngnathid fishes than in the other teleosts (*P* < 0.01; Fig. 8).

## Discussion

### Molecular characteristics of syngnathid mitogenomes

The mitochondrial genome organization of syngnathid fishes was quite conserved, as identified for other bony fishes [22, 31–33]. The differences in length among the Syngnathidae mitochondrial genome sequences are mainly due to length variation of the control region and random insertions in the intergenic regions. It is widely accepted that the mitochondrial DNA control region evolves faster than protein-coding genes [34, 35], and the pressure of purifying selection in the non-coding regions tends to be substantially weaker than that in coding regions [36]. Therefore, length variation would be more readily accumulated in the control region than in coding regions of the mitochondrial genome. In addition, random insertions of non-coding sequences between the mitochondrial genes were found in *T. serratus* and *C. flavofasciatus*, and these can be used as a basis for species identification. The insertion of non-coding sequences in the mitochondrial genome was also found in *Culicoides* species [37].

The mitochondrial nucleotide diversity of syngnathid fishes exhibited a similar pattern to that in other bony fishes, and the differences in sequence variability among mitochondrial genes will provide insight into their suitability for phylogenetic studies at various taxonomic levels [38, 39]. The codon usage bias was a textbook example of a weak selective pressure operating at the molecular level, and other evolutionary forces might explain its variation across different biological groups [40–42]. The two most frequently used codons in syngnathid fishes were different from those in other teleosts, and this may reflect a different evolutionary process for the mitochondrial genes of syngnathids compared to those of other teleosts. A significant bias towards A/T was observed in the codon usage of the mitochondrial genomes of syngnathids and other teleosts that may have contributed to the high A + T content in the mitogenome. However, codons in fish nuclear DNA sequences end predominantly in G or C, even though the coding sequences are not enriched in these nucleotides [43]. We found that G was the least common third position nucleotide in all the codon families in the mitochondrial sequences. We note that the abovementioned features are very similar to those observed in other vertebrates [44, 45].

### Phylogeny of syngnathid fishes

Syngnathids are perhaps the most unusual and specialized group of fishes considering their male reproductive mode [13]. The variation in pouch structure is one of the most important phenomena to have occurred throughout the evolutionary divergence of syngnathids [14]. The estimates of divergence times obtained in this study provide new information on the evolutionary history of syngnathids that can improve our understanding of the biological adaptability of pouch structure variation. Molecular clocks are vital for reconstructing detailed timescales in the tree of life that can explain how evolutionary events have been influenced by Earth’s history [46]. Molecular dating analyses indicated that the Syngnathidae most likely evolved 48.8 Mya, and this is concordant with the oldest known syngnathid fossil (48–50 Mya) [16, 47]. Our results suggested that the divergence of Syngnathidae likely resulted from global climate change during the late Paleocene and early Eocene. The Paleocene-Eocene thermal maximum was a short interval of maximum temperatures lasting approximately 100,000 years during the late Paleocene and early Eocene epochs (roughly 55 Mya) [48, 49]. Sea surface and continental air temperatures increased by more than 5 °C at that time, and this may have had a large impact on marine fishes, especially syngnathids, considering their distinct life-history characteristics and breeding strategies. Thus, we suspect that syngnathids experienced an adaptive radiation process during their early explosion of species. *Hippocampus reidi* and *H. ingens* diverged 3.7 Mya; the calibration point that we used in this study was based on evidence from a seahorse phylogeny that indicated that the closure of the Central American Seaway during the Late Pliocene resulted in the divergence of *H. reidi* (West Atlantic) and *H. ingens* (East Pacific) from a common ancestor (3.1–3.7 Mya) [50]. The results estimated using molecular clock approaches are consistent with the divergence events in the seahorse phylogeny.

The genus *Hippocampus* has evolved the most complex and advanced fully enclosed brood pouches for the delivery of nutrients and oxygen to the developing embryos [4, 12]. The molecular phylogeny obtained in this study shows that the development of brood pouch was an important evolutionary innovation of the Syngnathidae and the diversification of pouch types from simple ventral gluing areas to the completely enclosed pouches emphasizes the importance of the brood pouch structure variation in the radiation of syngnathids [15, 51, 52].

### Selection patterns within syngnathid mitogenomes

Nonsynonymous substitutions are generally harmful because they can cause defects in the respiratory electron transport chain and other metabolic processes [53, 54]. The conserved mitochondrial protein-coding sequences of syngnathid fishes might have undergone strong purifying selection to eliminate deleterious mutations. Syngnathid fishes appear to exhibit depressed *Ka/Ks* ratios for their mitochondrial OXPHOS genes compared with other teleostean fishes; this suggests that syngnathid fishes experience stronger selective constraints. Differential selection was also found in similar analyses performed in other fishes, birds, and mammals [27, 28, 30]. A study on mitogenome selection pressure in birds showed that stronger selective constraints act on highly locomotive birds and mammals with differing locomotive speeds exhibit a similar pattern [28]. Among fish species, the mitochondrial protein-coding genes of migratory fishes exhibit significantly lower *Ka/Ks* values than those of nonmigratory fishes [30]. The OXPHOS genes may have undergone stronger purifying selection because they play more important roles in energy metabolism than other mitochondrial genes. Moreover, the low number of SSRs in the mitochondrial coding regions of syngnathid fishes may indicate that their mitochondrial genomes are under strong purifying selection.

## Conclusions

The mitochondrial genome of eight syngnathid fishes were sequenced in this study, and molecular dating analyses indicated that the Syngnathidae most likely evolved 48.8 Mya, which is concordant with the oldest known syngnathid fossil. Syngnathid mitochondrial genes appear to exhibit depressed Ka/Ks ratios compared with those of other teleosts, and this may suggest that their mitogenomes have experienced strong selective constraints to eliminate deleterious mutations.

## Methods

### Source of data and primary treatment

The complete mitochondrial genome sequences of 88 teleost fishes available in September 2018 were downloaded from GenBank (Additional file 5). Additionally, the mitochondrial genomes of eight syngnathid fishes were newly sequenced for this study (*Trachyrhamphus serratus,* KJ184528; *Solegnathus hardwickii,* KJ184524; *Syngnathoides biaculeatus,* KJ184525; *Doryichthys boaja,* KJ184527; *Doryrhamphus dactyliophorus,* KP301502; *Microphis manadensis,* KP301501; *Hippocampus kelloggi,* KF703755; and *Hippocampus mohnikei,* KF557651). For the species to be newly sequenced, adults were collected from coastal areas of China in 2014–2017 (Fig. 1). A small amount of dorsal fin was sampled from every fish, which has no effect on the swimming and health, and then they were returned alive to the water. All samples used in this study were treated in accordance with relevant national and international guidelines.

Total genomic DNA was extracted from samples using the TIANGEN marine animal DNA kit (TIANGEN, Beijing, China) according to the manufacturer’s instructions. Degenerate primers for fragment amplification were designed based on conserved nucleotide sequences from the mitochondrial genomes of *Hippocampus kuda* (AP005985) and *Microphis brachyurus* (AP005986) using DNAssist 2.2 and Primer Premier 5.0 software [55]. The PCR amplifications, sequence assemble, and genome annotation were performed according to a slightly modified method described by Wang et al. [31].

### Phylogenetic analyses and molecular dating

All protein-coding genes were aligned using MAFFT version 7 [56]. The best-fitting nucleotide substitution model was selected using ModelTest 3.06 [57]. Maximum-likelihood (ML) analyses were implemented in PhyML 3.0 [58].

Molecular dating was performed using BEAST 1.4.6 [59]. A total of 26 calibration points was used in this analysis (Additional file 6). In each case, a normal prior was used, and its mean and standard deviation were set so that the 95% confidence intervals corresponded to the upper and lower bounds of each calibration point. Thus, uncertainty concerning the exact dates of the calibration points could be accounted for.

### Selection analyses

Comparison of the rates of nonsynonymous (*Ka*) and synonymous (*Ks*) substitutions can provide information on the type of selection that has acted on a given set of protein-coding sequences. The ratio of the rates of nonsynonymous to synonymous substitutions, ω (*Ka/Ks*), provides an indication of changes in selective pressure: *Ka/Ks* values > 1 indicate positive selection; *Ka/Ks* = 1 indicates neutral selection; and *Ka/Ks* < 1 indicates negative or purifying selection. The *Ka/Ks* ratios of all individual datasets were estimated for each branch of the phylogenetic tree using the CodeML algorithm from the PAML package [60]. The branch model was employed under two alternative assumptions: a one-ratio model, where one ω value was assumed for the entire tree, and a free-ratio model, where ω values were allowed to vary on every branch. We constructed a likelihood ratio test (LRT) to verify that the best models fitted the data. The level of significance for these LRTs was calculated using a χ^2^ approximation, where twice the difference in log likelihood between the models would follow a χ^2^ distribution, with the number of degrees of freedom corresponding to the difference in the number of parameters between the models.

### Comparative analysis of the mitochondrial genomes

MISA was used to analyze SSRs in the mitochondrial genomes [61]. SSRs were detected in the mitogenome sequences of 22 syngnathids and 22 other teleosts. The nucleotide diversity of the mitochondrial protein-coding gene sequences was evaluated using sliding window analysis based on the dataset used for SSR detection (window size = 200 bp, step size = 10 bp) in DnaSP version 5.10 [62]. The base composition was calculated using BioEdit version 7.1.3.0, and the AT-skew and GC-skew were calculated according to the formulae: AT-skew = (A − T%)/(A + T%) and GC-skew = (G − C%)/(G + C%). Relative synonymous codon usage (RSCU) of all protein-coding genes was analyzed using MEGA version 6.06 [63].

## Additional files

Additional file 1: List of the SSRs in the mitochondrial genome of 44 teleost fishes. (DOCX 27 kb)

Additional file 2: Statistics of the codon usage in the mitochondrial genes. (XLSX 13 kb)

Additional file 3: 95% confidence interval of dating analysis. (JPG 797 kb)

Additional file 4: Selection analysis of the mitochondrial genes. (XLSX 53 kb)

Additional file 5: List of the species used in this study. (XLSX 15 kb)

Additional file 6: Calibration points used in the divergence time analysis by BEAST List of the species used in this study. (XLSX 10 kb)

## Abbreviations

BP: Bootstrap probability
D-loop: Displacement loop
LRT: Likelihood ratio test
ML: Maximum-likelihood
RSCU: Relative synonymous codon usage
SSRs: Simple sequence repeats
