# Supplementary material for: Complete mitochondrial genomes of eight seahorses and pipefishes (Syngnathiformes: Syngnathidae): insight into the adaptive radiation of syngnathid fishes
Source: BMC Evol Biol. 2019 Jun 11;19:119. doi: 10.1186/s12862-019-1430-3 (PMC6560779; doi:10.1186/s12862-019-1430-3)
Supplement: Supplementary file 1 — List of the SSRs in the mitochondrial genome of 44 teleost fishes. (DOCX 27 kb) [file 12862_2019_1430_MOESM1_ESM.docx]

**Table 1** SSRs in mitogenomes of 44 fishes

| Species | SSR | start | end | Species | SSR | start | end |
| --- | --- | --- | --- | --- | --- | --- | --- |
| *Corythoichthys­-*  *flavofasciatus* | (CCGT)3 | 3246 | 3257 | *Aulorhynchus flavidus* | (TTC)4 | 14869 | 14880 |
|  | (CCTC)3 | 5971 | 5982 |  | (T)20 | 16303 | 16322 |
|  | (C)13 | 16528 | 16540 | *Lampetra fluviatilis* | (ATA)4 | 2048 | 2059 |
| *Doryichthys boaja* | (AAAT)3 | 1871 | 1882 |  | (CCAA)3 | 6723 | 6734 |
|  | (AT)9 | 15644 | 15661 |  | (T)12 | 12084 | 12095 |
|  | (TA)8 | 16443 | 16458 | *Myxine glutinosa* | (TTTAC)3 | 6353 | 6367 |
| *Doryrhamphus japonicus* | (AT)6 | 15658 | 15669 | *Fistularia commersonii* | (CCA)4 | 14186 | 14197 |
| *Dunckerocampus-*  *dactyliophorus* | (CCT)4 | 13036 | 13047 | *Gadus chalcogrammus* | (CTCCT)3 | 3045 | 3059 |
|  | (AT)8 | 15656 | 15671 |  | (CCCT)3 | 11599 | 11610 |
|  | (TA)6 | 16477 | 16488 |  | (T)13 | 15764 | 15776 |
| *Hippocampus kuda* | (AT)7 | 15668 | 15681 | *Harpadon microchir* | (TCC)4 | 5772 | 5783 |
| *Hippocampus barbouri* | (TCC)4 | 5788 | 5799 | *Hypoptychus dybowskii* | (CTTT)3 | 12068 | 12079 |
|  | (TA)8 | 15664 | 15679 | *Latimeria chalumnae* | (ACT)4 | 10217 | 10228 |
|  | (AAT)4 | 16209 | 16220 | *Lophius americanus* | (CCACC)3 | 1149 | 1163 |
| *Hippocampus comes* | (TA)8 | 15663 | 15678 |  | (T)12 | 16152 | 16163 |
|  | (AAT)4 | 16208 | 16219 | *Monopterus albus* | (CCT)4 | 11652 | 11663 |
| *Hippocampus erectus* | (CTAGGC)3 | 4073 | 4090 | *Myctophum affine* | (C)16 | 7388 | 7403 |
| *Hippocampus histrix* | (AT)7 | 15661 | 15674 |  | (C)12 | 14038 | 14049 |
| *Hippocampus ingens* | (TA)7 | 15667 | 15680 | *Neoceratodus forsteri* | (AAGT)3 | 14270 | 14281 |
| *Hippocampus kelloggi* | (AT)7 | 15677 | 15690 |  | (TA)6 | 16540 | 16551 |
| *Hippocampus mohnikei* | (CTT)4 | 14713 | 14724 | *Opistognathus jacksoniensis* | (TCC)4 | 8964 | 8975 |
|  | (AT)6 | 15662 | 15673 |  | (AACA)3 | 13521 | 13532 |
| *Hippocampus reidi* | (TA)7 | 15669 | 15682 | *Pagrus auriga* | (AACA)3 | 12411 | 12422 |
| *Hippocampus spinosissimus* | (AAAT)3 | 1530 | 1541 |  | (CTT)4 | 13657 | 13668 |
|  | (AT)7 | 15669 | 15682 | *Pagrus major* | (GGAA)3 | 1982 | 1993 |
| *Microphis brachyurus* | (AAAT)3 | 1876 | 1887 | *Pegasus volitans* | (ATCA)3 | 8228 | 8239 |
|  | (CAG)4 | 4209 | 4220 | *Percopsis transmontana* | (CTC)4 | 9027 | 9038 |
|  | (AT)8 | 15644 | 15659 |  | (CCT)4 | 10939 | 10950 |
|  | (ATTAT)5 | 16428 | 16452 | *Platax teira* | (AACA)3 | 13497 | 13508 |
| *Microphis manadensis* | (TA)8 | 15646 | 15661 | *Plecoglossus altivelis* | (TCCC)3 | 3637 | 3648 |
| *Syngnathoides biaculeatus* | (AT)7 | 15602 | 15615 |  | (CTT)4 | 6075 | 6086 |
| *Syngnathus schlegelli* | (CTC)4 | 8867 | 8878 |  | (TCT)4 | 9087 | 9098 |
