# Supplementary figures and images for: Complete mitochondrial genomes of eight seahorses and pipefishes (Syngnathiformes: Syngnathidae): insight into the adaptive radiation of syngnathid fishes
Source: BMC Evol Biol. 2019 Jun 11;19:119. doi: 10.1186/s12862-019-1430-3 (PMC6560779; doi:10.1186/s12862-019-1430-3)

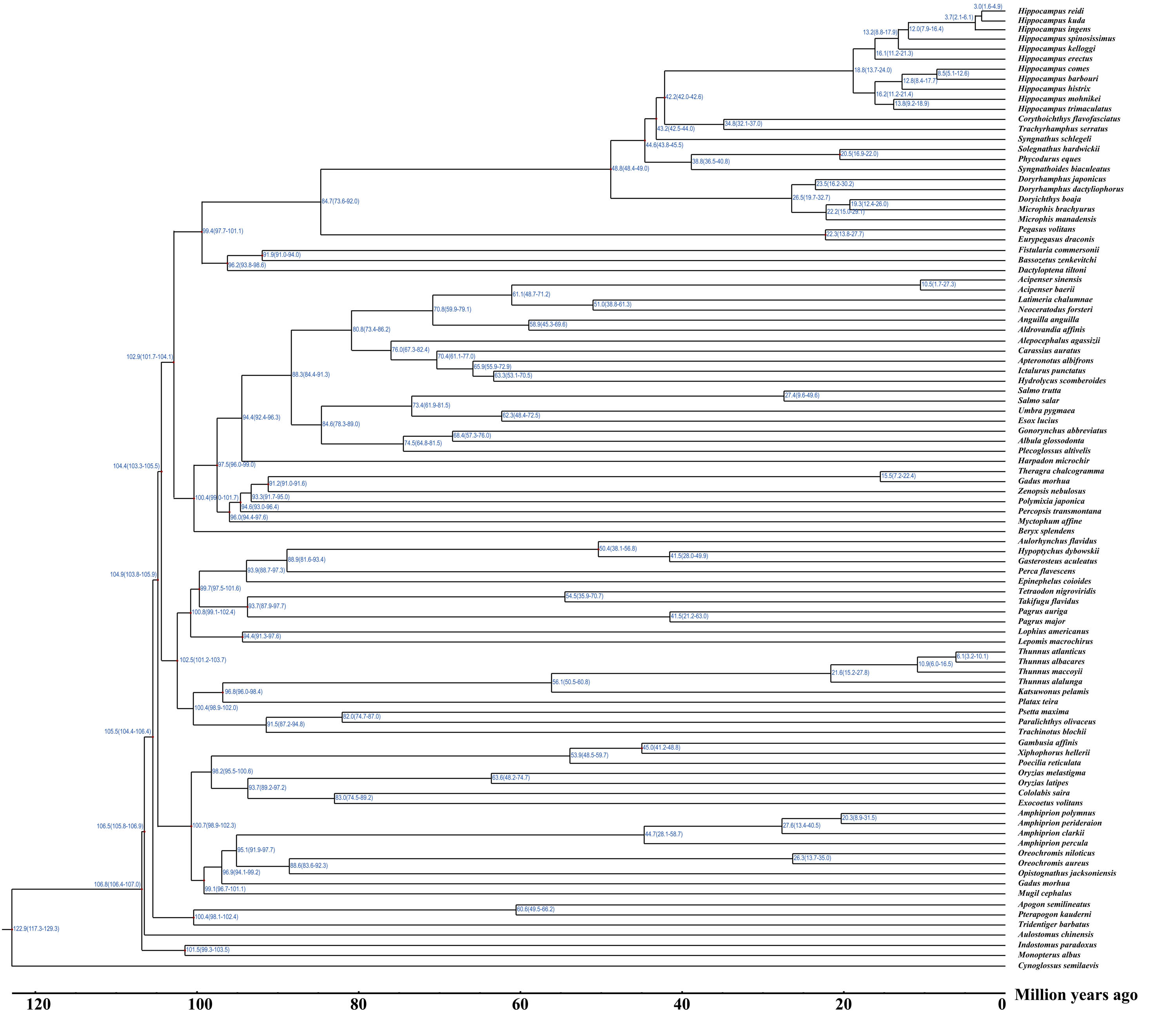

Supplement: Supplementary file 3 — 95% confidence interval of dating analysis. (JPG 797 kb) [file 12862_2019_1430_MOESM3_ESM.jpg]
